# Natriuretic Peptide Signaling *via* Guanylyl Cyclase (GC)-A: An Endogenous Protective Mechanism of the Heart

**DOI:** 10.2174/157340309787048068

**Published:** 2009-01

**Authors:** Ichiro Kishimoto, Takeshi Tokudome, Takeshi Horio, David L Garbers, Kazuwa Nakao, Kenji Kangawa

**Affiliations:** 1 National Cardiovascular Center, Research Institute 5-7-1 Fujishiro-dai Suita City Osaka 565-8565, Japan; 2 Howard Hughes Medical Institute, Department of Pharmacology, University of Texas, Southwestern Medical Center, Dallas, TX 75390-9051, USA; 3 Department of Medicine and Clinical Science, Kyoto University Graduate School of Medicine 54 Shogoin Kawaharacho Sakyou-ku Kyoto 606-8507, Japan

## Abstract

Atrial and brain natriuretic peptides (ANP and BNP, respectively) are cardiac hormones, secretions of which are markedly upregulated during cardiac failure, making their plasma levels clinically useful diagnostic markers. ANP and BNP exert potent diuretic, natriuretic and vasorelaxant effects, which are mediated *via* their common receptor, guanylyl cyclase (GC)-A (also called natriuretic peptide receptor (NPR)-A). Mice deficient for GC-A are mildly hypertensive and show marked cardiac hypertrophy and fibrosis that is disproportionately severe, given their modestly higher blood pressure. Indeed, the cardiac hypertrophy seen in these mice is enhanced in a blood pressure-independent manner and is suppressed by cardiomyocyte-specific overexpression of GC-A. These results suggest that the actions of a local cardiac ANP/BNP-GC-A system are essential for maintenance of normal cardiac architecture. In addition, GC-A was shown to exert its cardioprotective effects by inhibiting angiotensin II-induced hypertrophic signaling, and recent evidence suggests that regulator of G protein signaling (RGS) subtype 4 is involved in the GC-A-mediated inhibition of Gαq-coupled hypertrophic signal transduction. Furthermore, several different groups have reported that functional mutations in the promoter region of the human GC-A gene are associated with essential hypertension and ventricular hypertrophy. These findings suggest that endogenous GC-A protects the heart from pathological hypertrophic stimuli, and that humans who express only low levels of GC-A are genetically predisposed to cardiac remodeling and hypertension.

## INTRODUCTION

Normal cardiac structure is maintained by a sophisticated set of mechanical and cellular “checks and balances”, disturbance of which leads to a process called remodeling [1]. Although this process is initially adaptive, the beneficial effects are transient, and chronic cardiac remodeling leads to pathological molecular, cellular and interstitial changes that hinder cardiac function and ultimately lead to heart failure [2, 3]. Among the genes upregulated in cardiac remodeling, there are two that encode peptide hormones, atrial natriuretic peptide (ANP) [4, 5] and brain natriuretic peptide (BNP) [6]. ANP and BNP are synthesized, processed and secreted exclusively by the heart [7,8]. In response to the overactivation of various neurohumoral and mechanical stimuli that occur during heart failure, both the expression and secretion of ANP and BNP are dramatically upregulated, making their plasma levels clinically useful markers for the diagnosis and assessment of cardiac failure [9, 10]. Both natriuretic peptides exert potent diuretic, natriuretic and vasorelaxant effects through activation of their common receptor, guanylyl cyclase (GC)-A [also called natriuretic peptide receptor (NPR)-A] (Fig. (**1**)). GC-A is a prototype of plasma membrane-bound GCs, which serve as receptors that produce cyclic GMP (cGMP) in response to ligand binding [11]. C-type natriuretic peptide (CNP), the third member of the natriuretic peptide family, exerts its biological actions through another GC-coupled receptor called GC-B. In addition, the third receptor called clearance receptor mediates the metabolism of the natriuretic peptides. Because GC-A signaling stimulated by ANP and BNP results in a decrease in cardiac pre- and after-load, their mobilization during cardiac failure is thought to be one of the compensatory mechanisms activated in response to heart damage. In addition to the hemodynamic effects of their actions as circulating hormones, recent evidences suggest that ANP and BNP also act as autocrine/paracrine hormones. In the present review, we discuss the cardioprotective functions of endogenous ANP and BNP, focusing in particular on their local effects within the heart.

### *In Vitro* Studies Using Cultured Cardiomyocytes

The role of ANP as an autocrine factor involved in regulating cardiac myocyte growth is suggested by early *in vitro* studies carried out using cultured cardiomyocytes. 

Studies from Calderone *et al.* demonstrated the effects of exogenously applied ANP on heart cells [12]. Using cells cultured from neonatal rat heart, they observed that exogenously applied ANP caused concentration-dependent reductions in norepinephrine-stimulated incorporation of [^3^H]-leucine in myocytes and [^3^H]-thymidine in fibroblasts [12]. In both cell types, ANP increased intracellular cGMP levels, and 8-bromo-cGMP, a cGMP analogue, mimicked the growth-suppressing effects of ANP. In addition, ANP and 8-bromo-cGMP similarly attenuated the α1-adrenergic receptor-mediated increases in the mRNA level of proANP and decreases in the mRNA level of calcium ATPase in the sarcoplasmic reticulum (SR). The authors concluded that ANP diminishes the effects of norepinephrine on the growth of cardiac myocytes and fibroblasts, most likely* via* cGMP-mediated inhibition of the Ca^2+^ influx stimulated by norepinephrine.

In the opposite direction, we investigated the role of endogenously secreted ANP as an autocrine factor, treating cultured neonatal rat ventricular myocytes with HS-142–1, a non-selective receptor antagonist for GC-A and GC-B [13]. We found that the receptor antagonist increased both basal and phenylephrine-stimulated protein synthesis in a concentration-dependent manner, and these effects were accompanied by a significant increase in myocyte size. In addition, the expression of skeletal actin, β-myosin heavy chain and ANP, markers of hypertrophy, were elevated by treatment with the antagonist under both basal and phenylephrine-stimulated conditions. Conversely, both a cGMP-specific phosphodiesterase inhibitor, zaprinast, and a cGMP analogue suppressed basal and phenylephrine-stimulated protein synthesis. Thus, endogenous secretion of natriuretic peptides (ANP or BNP) from cardiomyocytes appears to inhibit cardiac myocyte hypertrophy under both basal and catecholamine-stimulated conditions, most likely* via* a cGMP-dependent process. 

These *in vitro* studies clearly suggest a local function of the natriuretic peptide system in the heart. However, since these studies were carried out using cultured neonatal cells, clarification of the physiological and pathophysiological effects of natriuretic peptides *in vivo* hearts was awaited for further investigation. In addition, although the results of cGMP and HS-142-1 implicated a role of GC-coupled receptors, it remained to be determined which receptor mediates the cardioprotective action of natriuretic peptides.

### *In Vivo* Studies Using the Mice Deficient for GC-A

To verify the hypothesis generated by the *in vitro* studies, the cardiovascular effects of endogenous natriuretic peptide signaling *in vivo* have been examined using the genetically engineered mice.

Because ANP and BNP share a common receptor (i.e., GC-A), the absence of one peptide can presumably be compensated for by the other. Therefore, to reveal the full effects of cardiac natriuretic peptide signaling, we generated mice that lacked endogenous GC-A (GC-A KO), and analyzed the cardiovascular phenotype [14-16]. As shown in Figs. (**2** and **3**), targeted deletion of the GC-A gene resulted in marked cardiac hypertrophy and fibrosis. Although GC-A KO mice also display mild hypertension, the cardiac hypertrophy was disproportionately severe, given the modest rise in blood pressure of the animal. In fact, other animal models that show similar increases in blood pressure do not exhibit the same degree of hypertrophy as GC-A KO mice [17, 18]. 

Independently, the research group led by Professors Smithies and Maeda at the University of North Carolina showed that mice lacking a functional Npr1 gene, which encodes NPR-A (i.e. GC-A), have elevated blood pressure and marked cardiac hypertrophy with interstitial fibrosis, resembling that seen in human hypertensive heart disease [19]. In their subsequent study, Knowles *et al.* reported that chronic treatment of GC-A KO with an ACE inhibitor, a diuretic, hydralazine or an angiotensin-receptor blocker, which all reduce blood pressure to the similar level as in wild-type mice, had no significant effect on the heart to body weight ratio [20]. Furthermore, in the reverse direction, pressure overload induced by transverse aortic constriction led to greater increases in ANP expression and in left ventricular weight to body weight ratio, in GC-A-KO mice than in wild-type mice [20]. Taken together, the authors concluded that the natriuretic peptide/GC-A system has direct antihypertrophic actions in the heart, independent of its role in blood pressure control. It is reported that targeted deletion of the proANP gene or proBNP gene also resulted in blood pressure-independent biventricular hypertrophy [21] or fibrosis [22], respectively, suggesting that a mechanism other than an increase in cardiac afterload exists for cardiac remodeling to be established in the mice lacking endogenous natriuretic peptides. In addition, since GC-A is expressed in the heart itself [23], it is suggested that this natriuretic peptide system acts in an autocrine/paracrine fashion to exert its cardioprotective effects. 

### Cardiomyocyte-Specific Overexpression or Deletion of GC-A Gene

Additional insights have also been gained from conditional overexpression or disruption of the GC-A gene. 

We generated transgenic mice in which the GC-A transgene was selectively overexpressed in cardiomyocytes [24]. Expression of this gene in the hearts of GC-A KO and wild-type mice did not alter blood pressure or heart rate in either group; however it did reduce the size of both normal myocytes in wild-type mice and hypertrophied myocytes in GC-A KO animals. Coincident with this reduction in myocyte size, cardiac levels of both mRNA and protein levels of ANP were significantly reduced. The genetic model thus separates the systemic regulation of cardiomyocyte size by blood pressure from the local regulation by myocardial effectors. 

The moderation of cardiac hypertrophy by local GC-A signaling was also demonstrated in a set of sophisticated experiments performed in the laboratory of Professor Kuhn at the University of Munich in Germany [25]. To test whether local ANP levels modulate cardiomyocyte growth, this group used homologous loxP/Cre-mediated recombination to selectively delete the GC-A gene in cardiomyocytes, thereby circumventing the systemic, hypertensive phenotype associated with germ line inactivation of GC-A. Mice with the cardiomyocyte-specific GC-A deletion exhibited mild cardiac hypertrophy and a marked increase in the transcription of cardiac hypertrophy markers. Blood pressure levels were 7-10 mmHg below normal, likely reflecting the endocrine actions of the systemically elevated ANP levels. On the other hand, in the mice, cardiac hypertrophic responses to aortic constriction were enhanced and accompanied by marked deterioration of cardiac function, indicating the significant role of cardiomyocyte GC-A in physiological as well as in pathophysiological conditions. 

Taken together, these results suggest that the natriuretic peptide system exerts a cardioprotective effect that is independent of its hemodynamic actions and implicate that activation of GC-A signaling has a direct inhibitory effect on cellular hypertrophic signaling within the heart [26].

### The Role of the Local Natriuretic Peptide System in Ang II-Induced Cardiac Remodeling

However, the precise mechanism by which activation of GC-A in cardiomyocytes protects the heart from excessive remodeling remained unclear. We previously demonstrated that cardiac hypertrophy and fibrosis in GC-A KO mice can be significantly diminished by targeted deletion of AT1a (double KO for GC-A and AT1a) or by pharmacological blockade of the receptor using a selective antagonist [27]. Conversely, stimulation of AT1a by exogenous application of Ang II, at a dose that does not affect blood pressure (subpressor dose), significantly exacerbated cardiac hypertrophy and dramatically augmented interstitial fibrosis in GC-A-KO mice, but not in wild-type animals. These results suggest that cardiac hypertrophy and fibrosis in GC-A-deficient mice are related, at least in part, to the enhanced cardiac AT1a signaling, and that endogenous GC-A inhibits the excessive activation of AT1a signaling.

### The Molecular Mechanism by which Cardiac GC-A Signaling Exerts its Cardioprotective Effect

To identify the differences between the hearts of GC-A KO and wild-type mice on a molecular level, we examined genes whose expression was upregulated in the hearts of GC-A KO mice and found that expression of the product of the Down syndrome critical region gene on chromosome 21, also called modulatory calcineurin-interacting protein 1 (MCIP1, more recently RCAN1), was increased. Because MCIP1 gene is upregulated by calcineurin signaling, we next investigated the role played by calcineurin in the cardiac hypertrophy of GC-A KO mice [28] and found that FK506-mediated blockade of calcineurin activation significantly reduced the heart weight to body weight ratio, cardiomyocyte size, and collagen volume fraction in GC-A KO mice, though FK506 had no effect on these parameters in wild-type mice. In cultured neonatal cardiomyocytes, pharmacological GC-A inhibition increased both basal and phenylephrine-stimulated calcineurin activities, while stimulation of GC-A by ANP inhibited these activities. We, therefore, suggest that it is by inhibiting calcineurin that cardiac GC-A signaling activated by locally secreted natriuretic peptides protects the heart from excessive cardiac remodeling [28]. To further explore the mechanism of the GC-A-mediated inhibition of calcineurin-induced hypertrophy, we assessed the G protein signaling that is known to occur upstream of calcium-calcineurin signaling and down-stream of Ang II signaling [29]. It was recently reported that cGMP-dependent protein kinase binds directly to and phosphorylates/activates regulator of G protein signaling subtype 2 (RGS2), which significantly increases the GTPase activity of Gα(q) and terminates G protein-coupled receptor signaling in vascular smooth muscle [30]. Given that cGMP is an intracellular second messenger for natriuretic peptides, we hypothesized that RGS might mediate the cardioprotective effect of GC-A signaling. To test that idea, we focused on RGS4, which is the dominant RGS in cardiomyocytes [29, 31]. In cultured cardiomyocytes, ANP stimulated the binding of cGMP-dependent protein kinase Iα  to RGS4, as well as the phosphorylation of RGS4 and its subsequent association with Gαq [29]. In addition, cardiomyocyte-specific overexpression of RGS4 in GC-A-KO mice significantly reduced the heart weight to body weight ratios, cardiomyocyte size and ventricular calcineurin activity. Conversely, overexpression of a dominant-negative form of RGS4 blocked the inhibitory effects of ANP on endothelin-1-stimulated inositol 1,4,5-triphosphate production, [^3^H]-leucine incorporation and ANP gene expression. These findings suggest that GC-A activates cardiac RGS4, which then inhibits the activity of Gαq and its downstream hypertrophic effectors, thereby playing a key role in the GC-A-mediated inhibition of cardiac hypertrophy. Fig. (**4**) shows a schematic diagram that depicts the endogenous cardio-protective mechanism meditated by ANP/BNP, GC-A and RGS4. 

## CLINICAL IMPLICATIONS

The experimental evidence obtained from the various animal models summarized above supports the notion that GC-A signaling plays a key role in the modulation of cardiac remodeling and blood pressure. Several groups have also investigated this relationship in human patients. For example, Nakayama *et al.* identified an 8 nucleotides insertion/deletion mutation at position -60 (60bp upstream of the ATG codon) in the 5'-flanking region (i.e., the promoter region) of the human NPR-A (GC-A) gene [32]. After genotyping 200 subjects with essential hypertension and 200 normotensive control subjects, they found nine individuals with a deletion that reduced the transcriptional activity of the GC-A promoter to less than 30% of the wild-type allele. All nine individuals were heterozygous for the allele and exhibited hypertension, left ventricular hypertrophy or both, suggesting that, in these individuals, this deletion reduces receptor expression and confers increased susceptibility to essential hypertension or left ventricular hypertrophy. In addition, Rubattu *et al.* investigated the relationships between ANP, BNP and GC-A polymorphisms and left ventricular structure in approximately 200 subjects with essential hypertension [33]. Those investigators identified ANP and NPR-A (GC-A) gene variants that were signifycantly associated with left ventricular mass index and left ventricular septal thickness. By contrast, BNP polymorphisms had no particular effect on cardiac phenotype. They concluded that the ANP/NPRA (GC-A) system contributed significantly to ventricular remodeling in human essential hypertension.

We also examined the association between polymorphisms within the GC-A promoter and essential hypertension in a group of Japanese subjects (177 hypertensive and 170 normotensive) and identified five allele types in which 6, 9, 10, 11 or12 CT dinucleotide repeats around position -293, upstream of the ATG codon [34]. The frequency of the (CT)n=6 allele was significantly higher among hypertensive subjects than among normotensive ones, while the frequencies of the other four allele types did not differ between the two groups. Promoter-reporter analyses carried out in cultured human aortic smooth muscle cells using a luciferase gene fused to the 5'-flanking region of the GC-A gene revealed that, like the promoter containing an 8bp deletion at position -60, the promoter containing (CT)n=6 at position -293 drove less transcriptional activity than the promoter containing (CT)n=10 (control) (Fig. (**5**)). Our results thus define the (CT)n polymorphism in the GC-A promoter as a potent and novel hypertension susceptibility marker. Although it did not reach statistical significance, preliminary data indicate that the incidence of left ventricular hypertrophy tends to be higher among patients carrying the (CT)n=6 allele than among those carrying other alleles.

Collectively, the findings summarized in this section suggest that people carrying the certain variants of human GC-A gene are more susceptible to cardiovascular diseases such as hypertension and cardiac hypertrophy than those who do not, possibly because they are resistant to the counter-regulation of Gαq signaling by ANP/BNP (Fig. (**6**)).

## CONCLUSION

Endogenous GC-A signaling protects the heart from excessive remodeling and failure. In addition to the improvement of cardiac pre- and after-load, it is suggested that GC-A-mediated local activation of RGS protein and subsequent suppression of Gαq hypertrophic signaling is involved in the action. Consequently, individuals who express only low levels of GC-A could be genetically prone to cardiac remodeling and hypertension.

## Figures and Tables

**Fig. (1):**
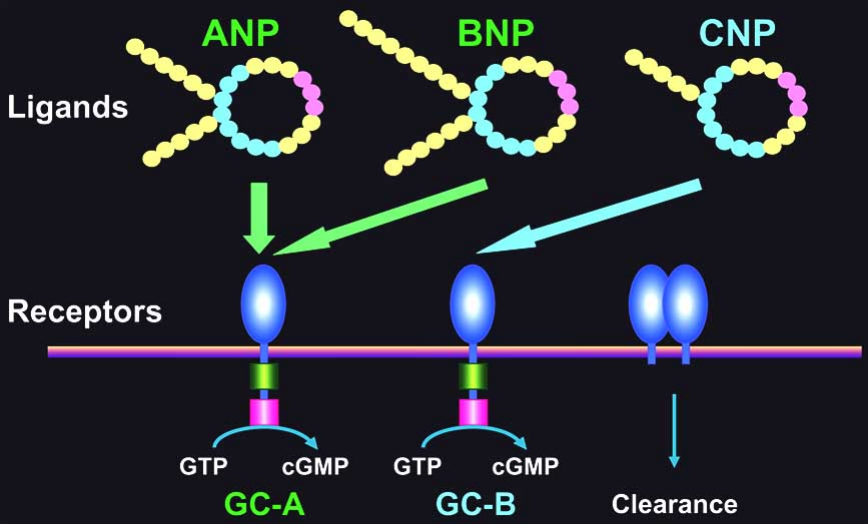
The mammalian natriuretic peptide system. The mammalian natriuretic peptide system is composed of at least three ligands (ANP, BNP and CNP) and three receptors (GC-A, GC-B and clearance receptor). ANP and BNP are cardiac hormones that are synthesized, processed and secreted by the heart. They exert their biological effects, which include diuresis, natriuresis and vasodilation, *via* a shared receptor, GC-A. ANP, atrial natriuretic peptide; BNP, brain natriuretic peptide; CNP, C-type natriuretic peptide; GC-A, guanylyl cyclase-A; GC-B, guanylyl cyclase-B.

**Fig. (2):**
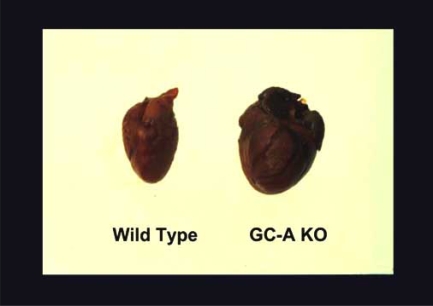
Cardiac Hypertrophy in GC-A KO. GC-A-deficient mice (GC-A KO) develop cardiac hypertrophy, as shown on the right.

**Fig. 3):**
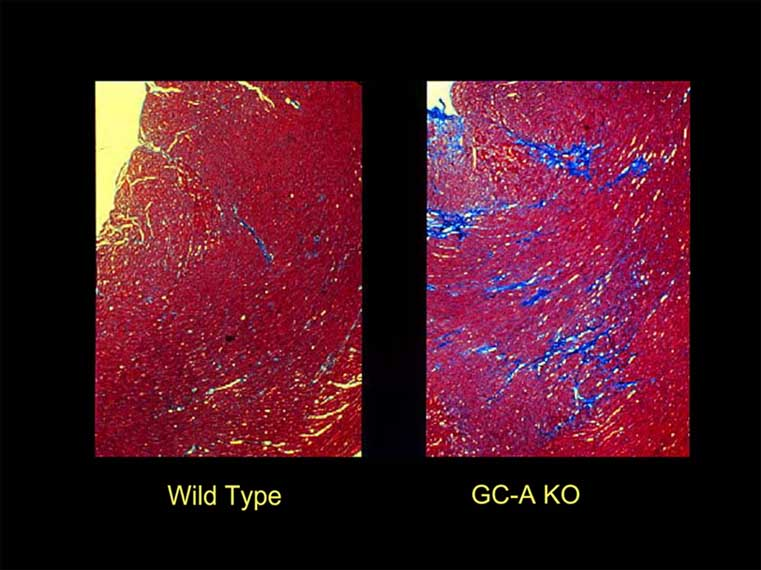
Cardiac Fibrosis in GC-A KO. GC-A-deficient (GC-A KO) mice develop cardiac fibrosis, as shown in right. Heart tissues were visualized after Masson-Trichrome staining. The fibrotic tissue is seen in blue.

**Fig. (4):**
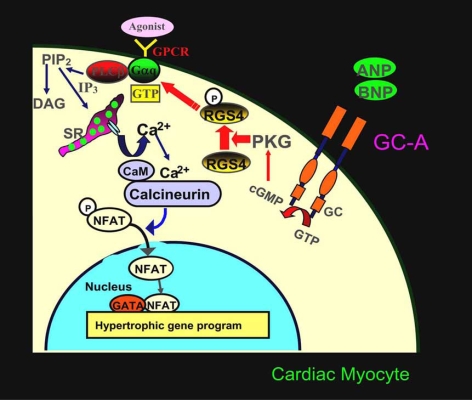
Schematic diagram depicting the pathway via which GC-A signaling inhibits cardiac hypertrophy. Cardiac hypertrophic agonists such as Ang II, norepinephrine and endothelin-1 stimulate G-protein-coupled receptors (GPCR) and activate phospholipase C (PLC). Subsequent production of inositol triphosphate (IP_3_) leads to release of Ca^2+^ from intracellular stores in the sarcoplasmic reticulum (SR), which raises cytosolic Ca^2+^ to a level sufficient to activate the calmodulin-regulated phosphatase calcineurin. Once activated, calcineurin dephosphorylates the transcription factor nuclear factor of activated T cells (NFAT), which facilitates its nuclear translocation. NFAT and GATA then act cooperatively to activate transcription of the hypertrophic gene program, including the ANP and BNP genes. We propose that endogenous ANP and BNP exert their antihypertrophic effects by stimulating GC-A/PKG-mediated regulator of G protein signaling subtype 4 (RGS4) phosphorylation/activation, which leads RGS4 to associate with Gαq, thereby increasing the GTPase activity of Gαq. This in turn inhibits calcineurin-NFAT signaling and suppresses hypertrophy-related gene transcription. Adopted from reference [29].

**Fig. (5):**
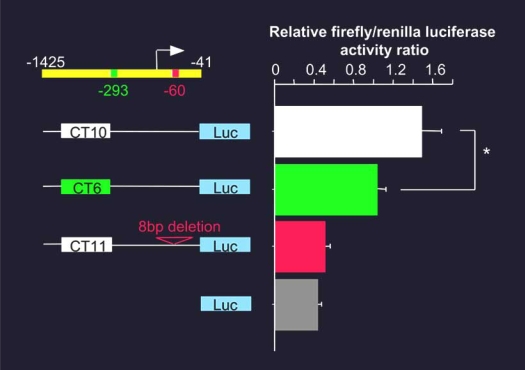
Transcriptional activities of the indicated (CT)n and 8-bp deletion reporter constructs in human aortic smooth muscle cells. Shown are the mean firefly (Luc)/renilla luciferase activity ratios. Note that the GC-A promoter containing (CT)n=6 or the 8bp deletion drove significantly less transcriptional activity than the promoter containing (CT)n=10. Modified from reference [34].

**Fig. (6):**
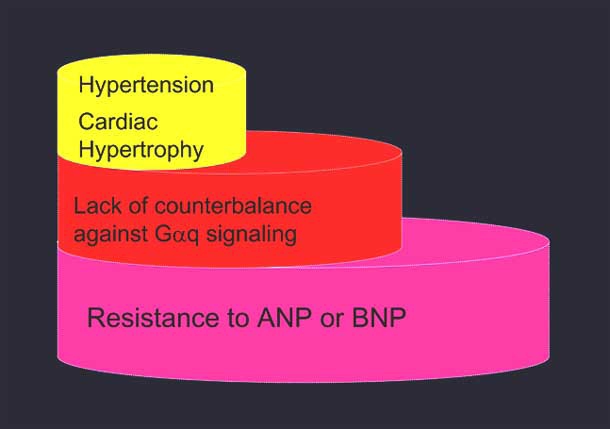
The patients with GC-A gene mutation are susceptible to cardiovascular diseases. These patients are resistant to ANP/BNP-mediated counter-regulation of Gαq signaling and are therefore more susceptible to cardiovascular diseases such as hypertension and cardiac hypertrophy.
